# Relationship between Telework Experience and Presenteeism during COVID-19 Pandemic, United States, March–November 2020

**DOI:** 10.3201/eid2902.221014

**Published:** 2023-02

**Authors:** Livvy Shafer, Faruque Ahmed, Sara Kim, Karen J. Wernli, Michael L. Jackson, Mary Patricia Nowalk, Todd Bear, Richard K. Zimmerman, Emily T. Martin, Arnold S. Monto, Manjusha Gaglani, Michael Reis, Jessie R. Chung, Brendan Flannery, Amra Uzicanin

**Affiliations:** Oak Ridge Institute for Science and Education, Oak Ridge, Tennessee, USA (L. Shafer);; Centers for Disease Control and Prevention, Atlanta, Georgia, USA (L. Shafer, F. Ahmed, S. Kim, J.R. Chung, B. Flannery, A. Uzicanin);; Kaiser Permanente Washington Health Research Institute, Seattle, Washington, USA (K.J. Wernli, M.L. Jackson);; University of Pittsburgh, Pittsburgh, Pennsylvania, USA (M.P. Nowalk, T. Bear, R.K. Zimmerman);; University of Michigan, Ann Arbor, Michigan, USA (E.T. Martin, A.S. Monto);; Baylor Scott and White Health, Temple, Texas, USA (M. Gaglani, M. Reis);; Texas A&M University College of Medicine, Temple (M. Gaglani, M. Reis)

**Keywords:** COVID-19, coronavirus disease, SARS-CoV-2, severe acute respiratory syndrome coronavirus 2, viruses, respiratory infections, zoonoses, pandemic, presenteeism, productivity, telework, United States

## Abstract

Persons with COVID-19–like illnesses are advised to stay home to reduce the spread of SARS-CoV-2. We assessed relationships between telework experience and COVID-19 illness with work attendance when ill. Adults experiencing fever, cough, or loss of taste or smell who sought healthcare or COVID-19 testing in the United States during March–November 2020 were enrolled. Adults with telework experience before illness were more likely to work at all (onsite or remotely) during illness (87.8%) than those with no telework experience (49.9%) (adjusted odds ratio 5.48, 95% CI 3.40–8.83). COVID-19 case-patients were less likely to work onsite (22.1%) than were persons with other acute respiratory illnesses (37.3%) (adjusted odds ratio 0.36, 95% CI 0.24–0.53). Among COVID-19 case-patients with telework experience, only 6.5% worked onsite during illness. Telework experience before illness gave mildly ill workers the option to work and improved compliance with public health recommendations to stay home during illness.

In response to the then-evolving COVID-19 pandemic, a public health emergency was declared in the United States on January 31, 2020, and several closure and containment policies were subsequently put in place. Those policies included restrictions on international and domestic travel, cancellation of public events, restrictions on gathering size, closure of schools and nonessential workplaces, and stay-at-home requirements (*1*). The prevalence of the closure and containment policies was greatest in April 2020 (*2*). The availability of COVID-19 testing was limited early in the pandemic but increased over time (*3*,*4*).

SARS-CoV-2 is spread through respiratory droplets produced by sneezing and coughing, as well as short-range and long-range aerosols (*5*). Shared workspaces, such as open office floor plans, shared offices, and break rooms, encourage the spread of SARS-CoV-2, thus making workplace transmission a concern. Employers in the United States were advised to implement a variety of measures to prevent and reduce transmission within the workplace, including symptom and temperature screening, mask wearing, physical distancing, increasing remote work where feasible, providing flexible paid sick leave, and actively encouraging sick employees to stay home (*6*). To support workers and businesses during the COVID-19 shutdown and reduce workplace spread of COVID-19, beginning April 1, 2020, the federal government provided direct financial support to workers and businesses and required covered employers to provide paid sick leave or expanded family and medical leave if an employee was unable to work because of COVID-19 illness (*7*). Nonetheless, outbreaks of COVID-19 in workplaces have been reported, in part attributed to presenteeism (*8*–*10*). Workplace presenteeism has been described as “the phenomenon of people, despite complaints and ill health that should prompt rest and absence from work, still turning up at their jobs” (*11*).

In a study of employed adults with influenza and other acute respiratory illnesses (ARIs) before the COVID-19 pandemic, Ahmed et al. (*12*) found that experience with telework before illness enabled workers to work more days overall during the first 3 days of illness than employees without telework experience. This experience enabled workers to maintain some level of workflow where they might otherwise have needed to use a sick day (*12*). Our study, conducted during the first year of the COVID-19 pandemic in the United States, examined whether telework experience before illness onset affected work attendance during illness.

## Methods

### Study Population

We enrolled adults 18–69 years of age seeking testing at COVID-19 testing sites or ambulatory medical care (i.e., telehealth, primary care, urgent care, or emergency department) for ARI (<10 days’ duration) manifesting as fever, cough, or loss of taste or smell. The sites were located in Michigan (Ann Arbor and Detroit), Pennsylvania (Pittsburgh), Texas (Temple and surrounding areas in Central Texas), and Washington (Puget Sound region) and were affiliated with the US Influenza Vaccine Effectiveness Network. Research staff screened persons for eligibility by telephone at 3 sites and by telephone or online survey at 1 site (Pennsylvania). The study methods have been published previously (*13*). This study was approved by institutional review boards at the Centers for Disease Control and Prevention and all participating sites. Study participants provided informed consent and were compensated $15–$20 for enrolling in the study.

### Data Collection

We collected data for this study during March 2020–November 2020, a period during which COVID-19 vaccines were not available in the United States. Participants completed an enrollment survey to provide information on their age, sex, race and ethnicity, education, occupation, general health status before illness, cigarette smoking or vaping, date of illness onset, and symptoms during illness. Persons who reported that they were healthcare workers might or might not have had close contact with patients. We tested respiratory specimens from midturbinate nasal swabs for SARS-CoV-2 by using reverse transcription PCR and recorded specimen collection and testing dates. Data on when participants received their COVID-19 test results were not available.

We asked participants to complete a follow-up survey 1–2 weeks after enrollment (Appendix Table 1). The follow-up survey included questions about employment status, hours expected to work in a typical week, hours usually worked from home (i.e., telework, telecommute, or remote work), and work status during illness (worked onsite, worked from home, did not work). We also asked participants about recovery from illness.

### Inclusion and Exclusion Criteria for Analysis

If an adult enrolled in the study >1 time because of multiple episodes of ARI, only the first enrollment was included. We excluded participants if they were unemployed, self-employed, owned their own business, worked solely from home before illness, or were employed for <20 hours per week. To minimize recall bias, we also excluded those who completed the follow-up survey >28 days after onset of illness.

### Definitions

We classified adults who reported that they regularly worked >1 hour from home in a typical week before their illness as having experience with telework (*12*). We computed duration of illness from the date of illness onset to the date of having fully or mostly recovered from illness. We categorized persons who reported working onsite for >1 day during their illness, regardless of whether they also teleworked during their illness, as having worked onsite during their illness. Among the remaining persons, we categorized those who reported teleworking for >1 day during their illness as having teleworked during illness (i.e., solely teleworked). We classified participants who did not fall into these 2 categories as not having worked during their illness. We computed days worked during illness by summing the days worked onsite and the days teleworked during illness. We defined laboratory-confirmed COVID-19 as a positive result from a PCR test.

### Statistical Analysis

We determined the associations between telework experience before illness and laboratory-confirmed COVID-19 with working onsite during illness to assess the potential to infect coworkers. We also assessed associations with working at all (onsite or solely telework) to assess maintenance of workflow. We used Student *t*-test to determine differences between means and χ^2^ test to assess differences between the observed frequencies and the expected frequencies if the null hypothesis was true. Wilcoxon’s rank-sum test was used to compare differences in spread and medians (*14*). We used PROC GLIMMIX in SAS version 9.4 (SAS Institute Inc., https://www.sas.com) to conduct multilevel logistic regressions to account for the clustering of participants within study sites. We determined covariables for inclusion in the models by using a backward selection process, assessing model fit using change in −2 log likelihood. Age, sex, health status, and smoking or vaping status were ultimately eliminated.

## Results

A total of 3,752 adults were enrolled across the study sites. Among 3,735 persons enrolled for the first time, 2,144 (57%) completed the follow-up survey within 28 days of illness onset. Survey completion rates were 22% (Michigan), 47% (Texas), 60% (Washington), and 80% (Pennsylvania). In total, 1,447 adults worked for an employer for >20 hours per week. After excluding persons who worked solely from home before illness, had missing information on telework experience before illness or work status during illness, had indeterminate or missing COVID-19 test results, had no symptoms, or had missing information for sex and healthcare personnel status, 947 persons were eligible (Figure).

**Figure:**
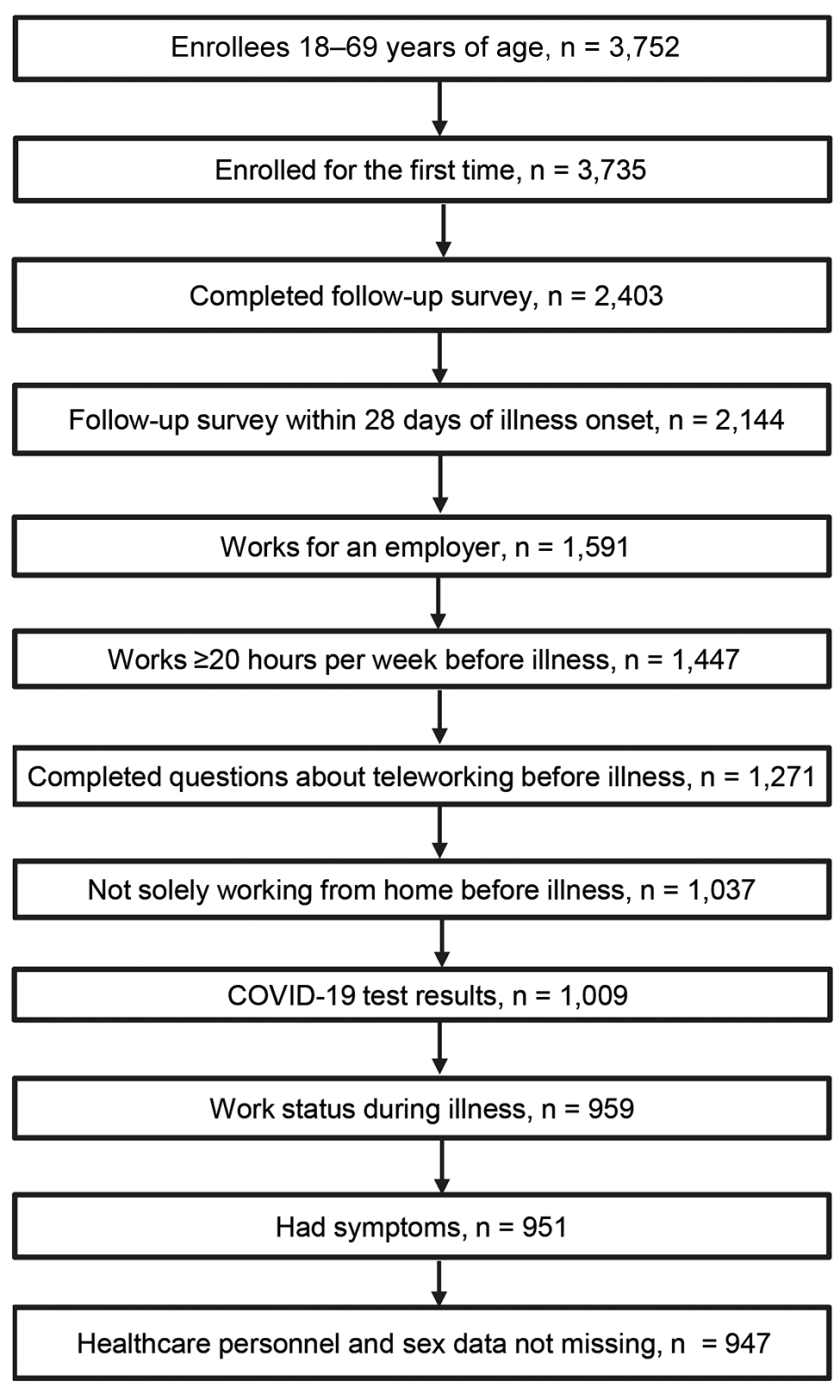
Enrollment flow diagram for adults seeking ambulatory medical care or testing at COVID-19 testing sites in study of relationship between telework experience and presenteeism during COVID-19 pandemic, United States, March–November 2020. Enrollment sites were in Michigan (Ann Arbor and Detroit), Pennsylvania (Pittsburgh), Texas (Temple and surrounding area in Central Texas), and Washington (Puget Sound region).

The median number of days from illness onset to specimen collection was 3 (interquartile range [IQR] 2–5) days, from illness onset to COVID-19 testing was 5 (IQR 3–8) days, from onset to enrollment was 6 (IQR 4–9) days, and from onset to follow-up was 15 (IQR 12–19) days. Enrollment occurred on the same day the specimen was collected in 28% of participants. Among the 947 participants, 231 (24.4%) persons had COVID-19 and 716 (75.6%) persons had non–COVID-19 ARIs. Compared with persons who did not have experience with telework before illness, those with telework experience were more likely to have illness onset during the fall than the spring (46.8% vs. 34.8%; p<0.01), more likely to have higher education levels (p<0.001), less likely to be a healthcare worker (36.6% vs. 52.7%; p<0.001), and less likely to have COVID-19 (15.1% vs. 27.0%; p<0.001) (Table 1).

**Table 1:** Characteristics of employed adults seeking ambulatory medical care or testing at COVID-19 testing sites, United States, March–November 2020*

Characteristic	Experience with telework before illness		p value
	Yes, n = 205	No, n = 742	
Site			<0.05†
Michigan	18 (8.8)	42 (5.7)	
Pennsylvania	101 (49.3)	437 (58.9)	
Texas	49 (23.9)	169 (22.8)	
Washington	37 (18.1)	94 (12.7)	
Illness onset period			<0.01†
Spring 2020, March–June	109 (53.2)	484 (65.2)	
Fall 2020, July–November	96 (46.8)	258 (34.8)	
Mean age, y (SD)	42.4 (11.1)	39.4 (11.6)	<0.001‡
Sex			<0.05†
M	69 (33.7)	188 (25.3)	
F	136 (66.3)	554 (74.7)	
Race/ethnicity			
White, non-Hispanic	169 (82.8)	590 (79.8)	
Black, non-Hispanic	6 (2.9)	60 (8.1)	
Other, non-Hispanic	14 (6.9)	39 (5.3)	
Hispanic, any race	15 (7.4)	50 (6.8)	
Education			<0.001†
Less than high school, high school diploma, or GED	5 (2.4)	92 (12.4)	
Some college, including vocational and associate's degree	32 (15.6)	285 (38.5)	
Bachelor's degree	68 (33.2)	214 (28.9)	
Advanced degree	100 (48.8)	149 (20.2)	
Healthcare personnel			<0.001†
No	130 (63.4)	351 (47.3)	
Yes	75 (36.6)	391 (52.7)	
Self-rated general health status			
Excellent	40 (19.5)	100 (13.5)	
Very good	89 (43.4)	308 (41.6)	
Good	61 (29.8)	270 (36.4)	
Fair/poor	15 (7.3)	63 (8.5)	
Smokes or vapes			
No	173 (84.4)	617 (83.2)	
Yes	32 (15.6)	125 (16.9)	
Median hours worked in a typical week before illness (10th, 90th percentile)	40 (36, 60)	40 (32, 48)	<0.001§
Fully or mostly recovered from illness at follow-up¶			<0.05†
No	22 (11.2)	124 (17.7)	
Yes	175 (88.8)	577 (82.3)	
Among persons fully or mostly recovered from illness, median duration of illness, d (10th, 90th percentile)	10 (4, 17)	10 (4, 17)	
COVID-19 case			<0.001†
Yes	31 (15.1)	200 (27.0)	
No	174 (84.9)	542 (73.1)	

*Values are no. (%) except as indicated. †By χ^2^ test. ‡By Student t test. §By Wilcoxon rank-sum test. ¶Data not available for 49 persons.

Persons with telework experience before illness were less likely to work onsite during their illness (21.5%) than those without telework experience (36.9%) (adjusted odds ratio [aOR] 0.45, 95% CI 0.30–0.68) (Table 2). COVID-19 case-patients were less likely to report working onsite during their illness (22.1%) than persons with non–COVID-19 ARIs (37.3%) (aOR 0.36, 95% CI 0.24–0.53) (Table 2). Among persons with telework experience before illness, only 6.5% of COVID-19 case-patients worked onsite (Table 2).

**Table 2:** Associations between telework experience before illness and COVID-19 status with working onsite during illness, United States, March–November 2020

Characteristic	Worked onsite during illness*		Adjusted odds ratio (95% CI)†
	Yes, n = 318	No, n = 629	
Telework experience before illness			
Yes	44 (21.5)	161 (78.5)	0.45 (0.30–0.68)
No	274 (36.9)	468 (63.1)	Referent
COVID-19 case			
Yes	51 (22.1)	180 (77.9)	0.36 (0.24–0.53)
No	267 (37.3)	449 (62.7)	Referent
Telework experience before illness: Yes			
COVID-19 case			
Yes	2 (6.5)	29 (93.6)	0.16 (0.03–0.82)
No	42 (24.1)	132 (75.9)	Referent
Telework experience before illness: No			
COVID-19 case			
Yes	49 (24.5)	151 (75.5)	0.38 (0.26–0.57)
No	225 (41.5)	317 (58.5)	Referent

*Among 318 persons categorized as having worked onsite during illness, 58 persons worked both onsite and remotely. Persons categorized as not having worked onsite during illness consisted of persons who did not work or solely teleworked. †Dependent variable in the multi-level logistic regression model is worked onsite during illness (0 = No, 1 = Yes). Independent variables are telework experience before illness (0 = No, 1 = Yes), COVID-19 case (0 = No, 1 = Yes), race/ethnicity, education, healthcare personnel status, hours typically worked per week before illness, illness onset period, and study site.

Persons with telework experience before illness were more likely to work at all (onsite or telework) during their illness (87.8%) than those with no telework experience (49.9%) (aOR 5.48, 95% CI 3.40–8.83) (Table 3). Among persons who worked, the median days worked was greater for those with telework experience than for those with no telework experience (>5 days vs. 3 days; p<0.001). Persons with COVID-19 were less likely to work at all during their illness (41.6%) than persons with non–COVID-19 ARIs (63.4%) (aOR 0.40, 95% CI 0.28–0.58) (Table 3).

**Table 3:** Associations between telework experience before illness and COVID-19 status with working at all during illness, United States, March–November 2020

Characteristic	Worked onsite or solely teleworked during illness*		Adjusted odds ratio (95% CI)†
	Yes, n = 550	No, n = 397	
Telework experience before illness‡			
Yes	180 (87.8)	25 (12.2)	5.48 (3.40–8.83)
No§	370 (49.9)	372 (50.1)	Referent
COVID-19 case			
Yes	96 (41.6)	135 (58.4)	0.40 (0.28–0.58)
No	454 (63.4)	262 (36.6)	Referent
Telework experience before illness: Yes			
COVID-19 case			
Yes	27 (87.1)	4 (12.9)	0.75 (0.22–2.61)
No	153 (87.9)	21 (12.1)	Referent
Telework experience before illness: No			
COVID-19 case			
Yes	69 (34.5)	131 (65.5)	0.39 (0.26–0.56)
No	301 (55.5)	241 (44.5)	Referent

*Persons categorized as having worked onsite includes persons who worked both onsite and teleworked. †Dependent variable in the multilevel logistic regression model is worked onsite or teleworked exclusively during illness (0 = No, 1 = Yes). Independent variables are telework experience before illness (0 = No, 1 = Yes), COVID-19 case (0 = No, 1 = Yes), race/ethnicity, education, healthcare personnel status, hours typically worked per week before illness, illness onset period, and study site. ‡Among the 550 persons who worked, the median days worked was ≥5 days for those with telework experience compared to 3 days for those with no telework experience (p<0.001). §Among 742 persons with no telework experience before illness, 117 (16%) teleworked during illness.

About 16% (75/467) of healthcare workers had telework experience before illness compared with 27% (130/481) of nonhealthcare personnel. The findings for working at all or onsite were similar in healthcare personnel and nonhealthcare personnel (Appendix Tables 2, 3). Findings for persons with illness onset during the spring were similar to those with illness onset during the fall (Appendix Tables 4, 5). Findings for the sites with higher survey completion rates (Pennsylvania and Washington) were similar to those for sites with lower survey completion rates (Michigan and Texas) (Appendix Tables 6, 7).

## Discussion

Our findings show that, during the early part of the COVID-19 pandemic, employed adults who had telework experience before becoming ill were less likely to work onsite but more likely to work at all (onsite or telework) during illness than those without telework experience, thus enabling them to maintain a greater level of productivity without risk of workplace-related onward spread of infection. Persons with COVID-19 were less likely to work at all, remotely or onsite, during illness than were persons with other ARIs. Among COVID-19 case-patients with telework experience before illness, few worked onsite when ill.

Persons with no telework experience might have been more likely to be in occupations that are less amenable to telework, and so they might not have had the option to work remotely instead of working onsite or using a sick day. For example, we found that healthcare personnel were less likely to telework than were nonhealthcare personnel. Previous studies reported that jobs in hospitality and leisure, transportation, utilities, production, agriculture, and construction are less likely to be amenable to telework than jobs in finance, law, computers, information, business and professional services, and many science fields (*15*,*16*).

Persons with COVID-19 might have been more likely to refrain from working during illness than those with non–COVID-19 ARIs for several reasons, including more, or more severe symptoms, such as fever, muscle aches, and a loss of smell or taste associated with COVID-19 illness (*13*); being advised to isolate by case investigators; and employers discouraging or prohibiting sick persons from entering the worksite. Our study found that 22% of persons with COVID-19 worked onsite, but that proportion was lower among persons with telework experience before illness. Some persons with COVID-19 might have worked onsite before they received a positive test result. Among persons with COVID-19 who had no telework experience, about one quarter worked onsite during illness, which is concerning because persons with COVID-19 are contagious for a prolonged period (*17*). Lockdown-style closures and containment measures were more prevalent in the spring of 2020 and COVID-19 testing was more available in the fall, but our findings were similar for these 2 periods (*3*,*4*).

A study in London found that approximately one third of persons with COVID-19 worked onsite while sick but did not assess the effect of experience with telework before illness (*18*). A study conducted before the COVID-19 pandemic during the 2017–18 influenza season found that persons with ARI or influenza who had telework experience before illness worked more days overall during illness than those without telework experience, although the number of days worked onsite was similar between the 2 groups (*12*). That study assessed work status during the first 3 days of illness, whereas our study assessed work status during illness over a longer period. In addition, 15% of participants had experience with telework before illness in the Ahmed et al. study, compared with 22% in our study. The proportion of persons who were excluded because of working solely from home was also lower in the Ahmed et al. study than in our study, which likely reflects the increased use of telework during the pandemic (*19*). Another study found that workers in Washington were significantly less likely to work while sick after the state passed a paid sick leave law (*20*), indicating a paid sick leave policy in combination with other nonpharmaceutical interventions could help reduce the spread of illness in the workplace.

A strength of our study was that participants were asked about frequency of telework before illness onset and during illness, enabling us to examine how telework experience before illness affected working onsite while sick. Another strength is the laboratory confirmation of COVID-19, which enabled us to distinguish those who had COVID-19 from those with other ARIs. Collecting sociodemographic data enabled us to control for variables such as race, ethnicity, and education.

The first limitation of our study is that a substantial number of persons did not complete follow up. It could be argued that nonrespondents were more likely to work onsite while sick. However, findings were similar for sites with higher follow-up rates (Pennsylvania and Washington) and those with lower follow-up rates (Michigan and Texas). Second, participants could have underreported working onsite because of the potential stigma around doing so in light of intensive public health messaging to stay home when ill. The comparisons between groups, however, would be valid if the underreporting was nondifferential. Third, we did not collect data on workplace policies, culture, and norms, and thus we are unable to assess how these factors affected our findings. Fourth, we do not know when participants learned the results of COVID-19 testing, so we could not assess what proportion of COVID-19 case-patients worked onsite before they were aware that they were SARS-CoV-2–positive. Fifth, we do not know when persons worked onsite during their illness. Persons who worked onsite later in the course of their illness might have been less infectious or even noninfectious. Finally, our findings might not be generalizable to asymptomatic persons, persons with milder symptoms who might not have sought medical care, or those with severe illness who were hospitalized for a prolonged period.

A systematic review published in 2019 found that the rate of persons going to work or school with an infectious illness ranged widely, from 35% to 97% (*21*). Presenteeism rates were generally higher in healthcare and social care workers (*21*). Reasons for presenteeism include lack of paid sick leave, a culture of presenteeism, potential disciplinary action, lack of coverage for work responsibilities, professionalism, job demands, concerns about being a burden on colleagues, colleagues’ perceptions, the threshold for absence because of sickness (i.e., illness was not severe and the person was well enough to work), and financial concerns. Future research should assess how workplace policies, culture, and occupations affect presenteeism in persons with COVID-19. Research should be done to quantify productivity in those who work during illness, as well as to assess the potential cost of infecting coworkers with illness if persons work onsite.

Our research demonstrates that telework experience before illness gave workers who were well enough the option to work during illness and improved compliance with the public health recommendation to stay home when ill. For jobs that are amenable to remote work, strategies should be developed to enable teleworking to become the norm for persons with ARIs.

Appendix Additional information about relationship between telework experience and presenteeism during COVID-19 pandemic, United States, March–November 2020
