# Supplementary material for: Relationship between Telework Experience and Presenteeism during COVID-19 Pandemic, United States, March–November 2020
Source: Emerg Infect Dis. 2023 Feb;29(2):278–85. doi: 10.3201/eid2902.221014 (PMC9881775; doi:10.3201/eid2902.221014)
Supplement: Appendix — Additional information about relationship between telework experience and presenteeism during COVID-19 pandemic, United States, March–November 2020 [file 22-1014-Techapp-s1.pdf]

Article DOI: https://doi.org/10.3201/eid2902.221014

# Relationship between Telework Experience and Presenteeism during COVID-19 Pandemic, United States, March–November 2020

## Appendix

**Appendix Table 1.** Follow-up survey questions, March-November 2020*

| Question | Values |
|---|---|
| Have you fully or mostly recovered from this illness? | Yes: date: (mm/dd/yyyy)<br>I have not fully or mostly recovered from this illness<br>Don’t know<br>Refused |
| Are you currently employed (work for pay or profit)? | I work for an employer<br>I am self-employed or own my own business [Survey is complete]<br>No [Survey is complete]<br>Refused [Survey is complete] |
| How many hours are you expected to work in a typical 7-day week? (If it varies, estimate the average.) | Hours<br>Don’t know<br>Refused<br>[If hours are less than 20, Don’t know, Refused, or Missing, survey is complete.] |
| Of those expected hours, how many hours in a week do you usually work from home (telework, telecommute, or remote work)? (Enter “0” if none) | Hours<br>Don’t know<br>Refused<br>[If expected hours equals hours worked from home, survey is complete.] |
| During your illness, how many days did you work on site? | 1, 2, 3, 4, ≥5 |
| Telework/work from home? | 1, 2, 3, 4, ≥5 |
| Not work? | 1, 2, 3, 4, ≥5 |

*Participants were asked to consider their primary job if they worked multiple jobs.

**Appendix Table 2.** Associations between telework experience before illness and COVID-19 status with working onsite during illness, by healthcare occupation, March–November 2020*

| | Worked onsite during illness | | |
|---|---|---|---|
| Characteristic | Yes, N = 318 | No, N = 629 | Adjusted odds ratio (95% CI)† |
| Healthcare personnel‡ | | | |
| Telework experience before illness | | | |
| Yes | 21 (28.0) | 54 (72.0) | 0.64 (0.35–1.18) |
| No | 150 (38.4) | 241 (61.6) | 1.00 (Referent) |
| Not healthcare personnel | | | |
| Telework experience before illness | | | |
| Yes | 23 (17.7) | 107 (82.3) | 0.36 (0.20–0.62) |
| No | 124 (35.3) | 227 (64.7) | 1.00 (Referent) |
| Healthcare personnel‡ | | | |
| COVID-19 case | | | |
| Yes | 30 (24.8) | 91 (75.2) | 0.38 (0.23–0.64) |
| No | 141 (40.9) | 204 (59.1) | 1.00 (Referent) |
| Not healthcare personnel | | | |
| COVID-19 case | | | |
| Yes | 21 (19.1) | 89 (80.9) | 0.31 (0.17–0.55) |
| No | 126 (34.0) | 245 (66.0) | 1.00 (Referent) |

*Values are no. (%) except as indicated. Among 318 persons categorized as having worked onsite during illness, 58 persons worked both onsite and teleworked. Persons categorized as not having worked onsite during illness comprised those who did not work or solely teleworked.
†Dependent variable in the multi-level logistic regression model is worked onsite during illness (0 = No, 1 = Yes). Independent variables are telework experience before illness (0 = No, 1 = Yes), COVID-19 case (0 = No, 1 = Yes), race/ethnicity, education, healthcare personnel status, hours typically worked per week before illness, illness onset period, and study site.
‡May or may not have close contact with patients.

**Appendix Table 3.** Associations between telework experience before illness and COVID-19 status with working at all during illness, by healthcare occupation, March–November 2020*

| | Worked onsite or solely teleworked during illness | | |
|---|---|---|---|
| Characteristic | Yes, N = 550 | No, N = 397 | Adjusted odds ratio (95% CI)† |
| Healthcare personnel‡ | | | |
| Telework experience before illness | | | |
| Yes | 65 (86.7) | 10 (13.3) | 6.21 (2.89–13.32) |
| No | 182 (46.6) | 209 (53.5) | 1.00 (Referent) |
| Not healthcare personnel | | | |
| Telework experience before illness | | | |
| Yes | 115 (88.5) | 15 (11.5) | 5.39 (2.88–10.08) |
| No | 188 (53.6) | 163 (46.4) | 1.00 (Referent) |
| Healthcare personnel‡ | | | |
| COVID-19 case | | | |
| Yes | 50 (41.3) | 71 (58.7) | 0.45 (0.27–0.73) |
| No | 197 (57.1) | 148 (42.9) | 1.00 (Referent) |
| Not healthcare personnel | | | |
| COVID-19 case | | | |
| Yes | 46 (41.8) | 64 (58.2) | 0.37 (0.22–0.61) |
| No | 257 (69.3) | 114 (30.7) | 1.00 (Referent) |

*Values are no. (%) except as indicated. Persons categorized as having worked onsite includes persons who worked both onsite and teleworked.
†Dependent variable in the multi-level logistic regression model is worked onsite or teleworked exclusively during illness (0 = No, 1 = Yes). Independent variables are telework experience before illness (0 = No, 1 = Yes), COVID-19 case (0 = No, 1 = Yes), race/ethnicity, education, healthcare personnel status, hours typically worked per week before illness, illness onset period, and study site.
‡May or may not have close contact with patients.

**Appendix Table 4.** Associations between telework experience before illness and COVID-19 status with working onsite during illness, by illness onset period, March–November 2020*

| Characteristic | Worked onsite during illness | | Adjusted odds ratio (95% CI)† |
|---|---|---|---|
| | Yes, N = 318 | No, N = 629 | |
| Spring 2020 (March–June) | | | |
| Telework experience before illness | | | |
| Yes | 21 (19.3) | 88 (80.7) | 0.35 (0.20–0.63) |
| No | 175 (36.2) | 309 (63.8) | 1.00 (Referent) |
| Fall 2020 (July–Nov) | | | |
| Telework experience before illness | | | |
| Yes | 23 (24.0) | 73 (76.0) | 0.50 (0.27–0.90) |
| No | 99 (38.4) | 159 (61.6) | 1.00 (Referent) |
| Spring 2020 (March–June) | | | |
| COVID-19 case | | | |
| Yes | 25 (22.7) | 85 (77.3) | 0.45 (0.27–0.76) |
| No | 171 (35.4) | 312 (64.6) | 1.00 (Referent) |
| Fall 2020 (July–Nov) | | | |
| COVID-19 case | | | |
| Yes | 26 (21.5) | 95 (78.5) | 0.29 (0.17–0.50) |
| No | 96 (41.2) | 137 (58.8) | 1.00 (Referent) |

*Values are no. (%) except as indicated. Among 318 persons categorized as worked onsite during illness, 58 persons worked both onsite and teleworked. Persons categorized as not worked onsite during illness comprised those who did not work or solely teleworked.
†Dependent variable in the multi-level logistic regression model is worked onsite during illness (0 = No, 1 = Yes). Independent variables are telework experience before illness (0 = No, 1 = Yes), COVID-19 case (0 = No, 1 = Yes), race/ethnicity, education, healthcare personnel status, hours typically worked per week before illness, illness onset period, and study site.

**Appendix Table 5.** Associations between telework experience before illness and COVID-19 status with working at all during illness, by illness onset period, March–November 2020*

| Characteristic | Worked onsite or solely teleworked during illness | | Adjusted odds ratio (95% CI)† |
|---|---|---|---|
| | Yes, N = 550 | No, N = 397 | |
| Spring 2020 (March–June) | | | |
| Telework experience before illness | | | |
| Yes | 91 (83.5) | 18 (16.5) | 3.55 (1.97–6.41) |
| No | 237 (49.0) | 247 (51.0) | 1.00 (Referent) |
| Fall 2020 (July–Nov) | | | |
| Telework experience before illness | | | |
| Yes | 89 (92.7) | 7 (7.3) | 10.00 (4.21–23.74) |
| No | 133 (51.6) | 125 (48.4) | 1.00 (Referent) |
| Spring 2020 (March–June) | | | |
| COVID-19 case | | | |
| Yes | 48 (43.6) | 62 (56.4) | 0.61 (0.39–0.97) |
| No | 280 (58.0) | 203 (42.0) | 1.00 (Referent) |
| Fall 2020 (July–Nov) | | | |
| COVID-19 case | | | |
| Yes | 48 (39.7) | 73 (60.3) | 0.24 (0.14–0.42) |
| No | 174 (74.7) | 59 (25.3) | 1.00 (Referent) |

*Values are no. (%) except as indicated. Persons categorized as worked onsite includes persons who worked both onsite and teleworked.
†Dependent variable in the multi-level logistic regression model is worked onsite or teleworked exclusively during illness (0 = No, 1 = Yes). Independent variables are telework experience before illness (0 = No, 1 = Yes), COVID-19 case (0 = No, 1 = Yes), race/ethnicity, education, healthcare personnel status, hours typically worked per week before illness, illness onset period, and study site.

**Appendix Table 6.** Associations between telework experience before illness and COVID-19 with working onsite during illness, by survey completion rate, March–November 2020*

| Characteristic | Worked onsite during illness | | Adjusted odds ratio (95% CI)† |
|---|---|---|---|
| | Yes, N = 318 | No, N = 629 | |
| Survey completion rate of 76%‡ | | | |
| Telework experience before illness | | | |
| Yes | 32 (23.2) | 106 (76.8) | 0.65 (0.40–1.04) |
| No | 183 (34.5) | 348 (65.5) | 1.00 (Referent) |
| Survey completion rate of 42%§ | | | |
| Telework experience before illness | | | |
| Yes | 12 (17.9) | 55 (82.1) | 0.14 (0.06–0.33) |
| No | 91 (43.1) | 120 (56.9) | 1.00 (Referent) |
| Survey completion rate of 76%‡ | | | |
| COVID-19 case | | | |
| Yes | 35 (21.5) | 128 (78.5) | 0.37 (0.23–0.58) |
| No | 180 (35.6) | 326 (64.4) | 1.00 (Referent) |
| Survey completion rate of 42%§ | | | |
| COVID-19 case | | | |
| Yes | 16 (23.5) | 52 (76.5) | 0.22 (0.11–0.48) |
| No | 87 (41.4) | 123 (58.6) | 1.00 (Referent) |

*Values are no. (%) except as indicated. Among 318 persons categorized as worked onsite during illness, 58 persons worked both onsite and teleworked. Persons categorized as not worked onsite during illness comprised those who did not work or solely teleworked.
†Dependent variable in the multi-level logistic regression model is worked onsite during illness (0 = No, 1 = Yes). Independent variables are telework experience before illness (0 = No, 1 = Yes), COVID-19 case (0 = No, 1 = Yes), race/ethnicity, education, healthcare personnel status, hours typically worked per week before illness, illness onset period, and study site.
‡Pennsylvania and Washington combined.
§Michigan and Texas combined.

**Appendix Table 7.** Associations between telework experience before illness and COVID-19 with working at all during illness, by survey completion rate, March–November 2020*

| Characteristic | Worked onsite or solely teleworked during illness | | Adjusted odds ratio (95% CI)† |
|---|---|---|---|
| | Yes, N = 550 | No, N = 397 | |
| Survey completion rate of 76%‡ | | | |
| Telework experience before illness | | | |
| Yes | 122 (88.4) | 16 (11.6) | 4.91 (2.75–8.76) |
| No | 274 (51.6) | 257 (48.4) | 1.00 (Referent) |
| Survey completion rate of 42%§ | | | |
| Telework experience before illness | | | |
| Yes | 58 (86.6) | 9 (13.4) | 6.80 (2.81–16.47) |
| No | 96 (45.5) | 115 (54.5) | 1.00 (Referent) |
| Survey completion rate of 76%‡ | | | |
| COVID-19 case | | | |
| Yes | 74 (45.4) | 89 (54.6) | 0.46 (0.31–0.68) |
| No | 322 (63.6) | 184 (36.4) | 1.00 (Referent) |
| Survey completion rate of 42%§ | | | |
| COVID-19 case | | | |
| Yes | 22 (32.4) | 46 (67.7) | 0.21 (0.10–0.43) |
| No | 132 (62.9) | 78 (37.1) | 1.00 (Referent) |

*Values are no. (%) except as indicated. Persons categorized as worked onsite includes persons who worked both onsite and teleworked.
†Dependent variable in the multi-level logistic regression model is worked onsite or teleworked exclusively during illness (0 = No, 1 = Yes). Independent variables are telework experience before illness (0 = No, 1 = Yes), COVID-19 case (0 = No, 1 = Yes), race/ethnicity, education, healthcare personnel status, hours typically worked per week before illness, illness onset period, and study site.
‡Pennsylvania and Washington combined.
§Michigan and Texas combined.
